# Targeted isolation, sequence assembly and characterization of two white spruce (*Picea glauca*) BAC clones for terpenoid synthase and cytochrome P450 genes involved in conifer defence reveal insights into a conifer genome

**DOI:** 10.1186/1471-2229-9-106

**Published:** 2009-08-06

**Authors:** Björn Hamberger, Dawn Hall, Mack Yuen, Claire Oddy, Britta Hamberger, Christopher I Keeling, Carol Ritland, Kermit Ritland, Jörg Bohlmann

**Affiliations:** 1 Michael Smith Laboratories, University of British Columbia, 2185 East Mall, Vancouver, B.C., V6T 1Z4, Canada; 2 Department of Forest Sciences, University of British Columbia, Vancouver, B. C., V6T 1Z4, Canada

## Abstract

**Background:**

Conifers are a large group of gymnosperm trees which are separated from the angiosperms by more than 300 million years of independent evolution. Conifer genomes are extremely large and contain considerable amounts of repetitive DNA. Currently, conifer sequence resources exist predominantly as expressed sequence tags (ESTs) and full-length (FL)cDNAs. There is no genome sequence available for a conifer or any other gymnosperm. Conifer defence-related genes often group into large families with closely related members. The goals of this study are to assess the feasibility of targeted isolation and sequence assembly of conifer BAC clones containing specific genes from two large gene families, and to characterize large segments of genomic DNA sequence for the first time from a conifer.

**Results:**

We used a PCR-based approach to identify BAC clones for two target genes, a terpene synthase (3-carene synthase; 3CAR) and a cytochrome P450 (CYP720B4) from a non-arrayed genomic BAC library of white spruce (*Picea glauca*). Shotgun genomic fragments isolated from the BAC clones were sequenced to a depth of 15.6- and 16.0-fold coverage, respectively. Assembly and manual curation yielded sequence scaffolds of 172 kbp (3CAR) and 94 kbp (CYP720B4) long. Inspection of the genomic sequences revealed the intron-exon structures, the putative promoter regions and putative *cis*-regulatory elements of these genes. Sequences related to transposable elements (TEs), high complexity repeats and simple repeats were prevalent and comprised approximately 40% of the sequenced genomic DNA. An *in silico *simulation of the effect of sequencing depth on the quality of the sequence assembly provides direction for future efforts of conifer genome sequencing.

**Conclusion:**

We report the first targeted cloning, sequencing, assembly, and annotation of large segments of genomic DNA from a conifer. We demonstrate that genomic BAC clones for individual members of multi-member gene families can be isolated in a gene-specific fashion. The results of the present work provide important new information about the structure and content of conifer genomic DNA that will guide future efforts to sequence and assemble conifer genomes.

## Background

Conifers (*Coniferales*) are a large group of gymnosperm trees which are separated from the angiosperms by more than 300 million years of independent evolution. The conifers include the economically and ecologically important species of spruce (*Picea*) and pine (*Pinus*), which dominate many of the world's natural and planted forests [1]. The development of genomic resources for conifers has focused on the discovery and characterization of expressed genes in the form of expressed sequence tags (ESTs) and full-length (FL)cDNAs. The available conifer cDNA sequence resources are extensive (1,158,419 ESTs as of December 3, 2008), representing almost 9% of all ESTs in the plant genome database (http://plantgdb.org/, http://www.ncbi.nlm.nih.gov/dbEST/dbEST_summary.html). The EST and FLcDNA resources developed for white spruce (*Picea glauca*), Sitka spruce (*P. sitchensis*), and a hybrid white spruce (*P. glauca *× *P. engelmannii*) [2,3], have enabled transcriptome profiling [1,4-6], proteome analysis [7-9], marker development [10-13], and the functional characterization of gene products [14-16]. These functional genomics studies have provided considerable insights into conifer defence against insects and pathogens, adaptation to the environment, and development [1,4].

Beyond the characterization of cDNAs and their encoded proteins, the lack of a gymnosperm reference genome sequence limits our knowledge of the organization, structure and gene space of conifer genomes. Sequencing a conifer genome has not yet been attempted and will remain a daunting task, given that conifer genomes range in size from 20 to 40 Gbp, which is 200 - 400-fold larger than the genome of *Arabidopsis *and larger than any other genome sequenced to date. The sequencing of a conifer genome may also be challenging due to a very high content of repetitive DNA [17] and the tendency of conifers to out-cross, preventing the development of inbred strains. An important step in assessing the feasibility of conifer genome sequencing will be the isolation, in random or targeted fashion, of genomic (g)DNA in the form of BAC clones, followed by the sequencing and assembly of large segments of gDNA. However, to the best of our knowledge, sequencing of a complete BAC clone or any large segment of nuclear gDNA has not yet been reported in the literature for a conifer or any other gymnosperm species. Recently, a loblolly pine (*Pinus taeda*) gDNA BAC library was used to assess the contribution of a novel pine-specific retrotransposon family (Gymny) to conifer genome size [18].

Unlike in angiosperms, conifers are not thought to have undergone recent genome duplication events [17,19]. However, two features of conifer genomes pose untested challenges for the targeted isolation and sequence assembly of BACs containing genes of interest involved in conifer defence. First, many conifer defence genes exist as closely related members of large families. For example, genes encoding the oleoresin producing terpenoid synthases (TPSs) [14,15], cytochrome P450 monooxygenases (P450s) involved in diterpene resin acid formation (CYP720B) [20,21], TIR-NBS-LRR disease resistance proteins [22], pathogenesis-related (PR)-10 proteins [23], and dirigent proteins [24,25] are members of such multigene families. Against the background of large gene families it may be difficult to isolate BACs for a specific target gene. Second, the abundance of transposable elements (TEs), specifically those of the Copia and Gypsy classes, which have been demonstrated by *in situ *hybridizations as diverse families of retroelements across conifer chromosomes [26,27], may cause additional problems with genome sequence assemblies.

In this paper we report a successful strategy for the targeted BAC identification and isolation of TPS and P450 genes using PCR-based screening of a non-arrayed white spruce BAC library of 3X genome coverage, and the subsequent gDNA insert sequencing, sequence assembly, and sequence characterization. When extended to other conifers, our strategy will enable a comparative analysis of synteny of specific target regions of conifer genomes.

## Results

### Targeted isolation of BAC clones containing TPS (3CAR) and P450 (CYP720B4) genes

Our first objective was to test if individual BAC clones containing conifer genes of large gene families could be isolated in a gene-specific manner. A white spruce (genotype PG29) gDNA BAC library of approximately 3X genome coverage was constructed, aliquoted into pools in ten 96-well plates, and screened in a hierarchical fashion by PCR as described previously [28]. The primers used to screen pooled BAC clones for a specific TPS gene were based on the functionally characterized Norway spruce (*Picea abies*) and Sitka spruce 3-carene synthase FLcDNAs (3CAR) [[29], D. Hall, J. Robert, C.I. Keeling, J. Bohlmann, unpublished results]. Primers used to screen for a specific target P450 gene were based on the functionally characterized diterpene oxidase CYP720B4 from Sitka spruce and its white spruce orthologue [B. Hamberger, T. Ohnishi, J. Bohlmann, unpublished results]. The function of the spruce CYP720B4 gene is similar to that of loblolly pine CYP720B1 in diterpene resin acid formation [20,21]. Primers used for gene-specific screening for TPS (3CAR)- or P450 (CYP720B4)-containing BAC clones were assessed *in silico *against other known members of the large conifer TPS-d family [15] and other members of the conifer-specific CYP720B family [20], respectively, to minimize the chance of isolating non-target members of these gene families.

From a total of 960 BAC pools (ten 96-well plates), which were initially screened as 200 super-pools (20 super-pools per 96-well plate) we identified 23 and 18 pools that yielded PCR products with the 3CAR and CYP720B4 primers, respectively. The 23 independent PCR products obtained with 3CAR primers represented four unique 3CAR-like sequences with at least 95% identity (in the open reading frame) amongst each other and to the Sitka spruce 3CAR FLcDNA Q09 (see Additional file 1). We also sequenced five independent PCR products obtained by screening the BAC pools with CYP720B4 primers. All five sequences were 100% identical with the target CYP720B4 sequence. For each of the two target genes, a single individual BAC clone was isolated, verified by sequencing the PCR product, and the gDNA inserts were excised and their size estimated based upon their mobility in pulsed field gel electrophoresis. The BAC clone PGB02 (3CAR) contained a gDNA insert of approximately 185 kbp and BAC clone PGB04 (CYP720B4) contained an insert of approximately 110 kbp. These gDNA inserts were sheared into fragments of 700 - 2000 bp and shotgun-subcloned into plasmid libraries for sequencing.

### Automated sequence assemblies of PGB02 and PGB04

The shotgun plasmid libraries for PGB02 and PGB04 were arrayed in 384-well plates. Plasmid inserts from ten and five 384-well plates were Sanger-sequenced for PGB02 and PGB04, respectively, resulting in 6,954 and 3,677 paired sequence reads (see Additional file 2). The average plasmid insert length was 1,102 bp for the PGB02 library and 1,056 bp for the PGB04 library. Sequences were scanned and masked for vector sequences and contaminating bacterial sequences, eliminating 21.4% (PGB02) and 27.9% (PGB04) of the total sequences. Using PHRAP, we assembled the sequences into 15 contigs for PGB02 and 14 contigs for PGB04. For PGB02, the two largest contigs assembled in this automated fashion covered a total length of 172,403 bp (91.2% of the sequence reads); the three largest contigs for PGB04 covered over 93,905 bp (94.4% of the sequence reads) (see Additional file 3).

### Manual curation of the sequence assemblies of PGB02 and PGB04

**Figure 1:**
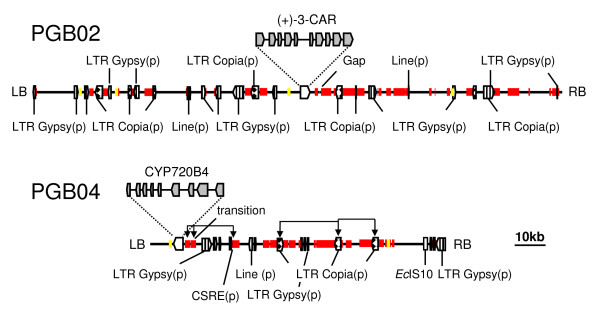
**Structure of white spruce genomic DNA of BAC clones PGB02 and PGB04**. The position of the target genes 3CAR and CYP720B4 is indicated. Red and yellow bars represent repeated segments and segments with similarity to DNA transposons, respectively. Transposable elements were identified with the RepeatMasker using the *viridiplantae *section of the RepBase Update database. EcIS10, *E. coli *individual insertion sequence (IS) of the bacterial transposon Tn*10*; CSRE, conifer specific repeat element; LB/RB left and right border of pINDIGO; arrows in PGB04 indicate local putative segment duplications. The scale bar represents 10 kbp. (p) pseudogene, based on the accumulation of deleterious mutations and the absence of transcript with >90% identity.

To improve the assembly of PGB02 and PGB04, we inspected each contig generated with the PHRAP software. We found that chimeric sequences, resulting from the ligation of independent gDNA fragments during the production of shotgun plasmid libraries, were included in some of the plasmid insert sequences, which together with low-quality sequences and low-complexity repeats, prevented the automated assembly into continuous sequence. In addition, we manually aligned shorter contigs with low sequence representation to the larger contigs. The left and right arms of the pIndigoBAC-5 vector, which were subcloned together with the gDNA inserts into the plasmid shotgun libraries, provided orientation for the scaffolds of PGB02 and PGB04 (Figure 1).

The final assembly of PGB02 contained two contigs separated by a short gap (approximately 25 - 50 bp based on PCR amplification of the gap region) without sequence coverage. The gap is flanked by long stretches of low-complexity repeat sequence. It is likely that the sequence gap resulted from physical repeat structures (e.g., hairpins) which interfered with sequencing this region. Manual curation resulted in a single complete contig for the PGB04 gDNA. In PGB04 two high-complexity repeats and several low-complexity repeats extend for over 1 kbp on either side of a region of approximately 200 bp with low sequence coverage (transition) (Figure 1).

In summary, the combined automated and manual sequence assemblies resulted in two contigs for PGB02 with a combined sequence length of 172,056 bp and 15.6× sequence coverage, and into a single contigs for PGB04 with a sequence length of 93,592 bp and 16.0× sequence coverage. The size of the assembled sequence contigs for PGB02 and PGB04 agree well with the size of BAC inserts as estimated by PFGE (185 kbp and 110 kbp, respectively).

### *In silico *analysis of the effect of sequencing depth on assembly quality

Using the high sequence coverage (16×) and high-quality manually curated sequence assembly (93,592 bp) for PGB04 we analyzed the effect of plasmid shotgun library sequencing depth on the quality of the automated assembly. This assessment can guide cost-effective sequencing of BAC clones for future efforts of conifer genome sequencing. The sequences obtained from the plasmids of five 384-well plates for PGB04 were assembled into independent builds in all permutations of two, three, four or five plates (see Additional files 4 and 5). With sequences obtained from one plate, an average coverage of 3.2× was obtained and the number of nucleotides assembled into contigs (average contig number of 22.2) was less than 90 kbp (representing 93.0% coverage). By assembling sequences from two plates, the coverage doubled to an average of 6.4×, the number of contigs (average 9.9) was reduced, the assembly included over 95 kbp in contigs, and the full length scaffold had over 98% coverage relative to the reference PGB04 assembly. When sequences from three, four or five plates were used in the assembly, coverage increased to 9.6×, 12.8× and 16×, respectively, with a further increase in the number of nucleotides assembled. The assembly of sequences from three, four or five plates also resulted in an increase of the number of contigs. Even with five plates, the coverage obtained by automated assembly never reached 100% relative to the PGB04 reference assembly, which involved manual curation.

### Gene content of PGB02 and PGB04

Results from the overall sequence analyses of the BAC clones PGB02 and PGB04, visualised using gbrowse, are available as online information at http://treenomix3.msl.ubc.ca/cgi-bin/gbrowse/PGB02/; http://treenomix3.msl.ubc.ca/cgi-bin/gbrowse/PGB04/ (username: treenomix; password: conifer). These descriptions include BLAST annotations (against NCBI NR, MIPS coniferales repeats, spruce ESTs), GC content and gene predictions [Genemark Prediction (Eukaryotic HMM), FGENESH Prediction, Genescan Prediction]. PGB02 and PGB04 each contained a single functional gene identified by BLAST searches, which match the target genes 3CAR (PGB02) and CYP720B4 (PGB04) (Figure 1). Relative to the complete gDNA sequence length of PGB02 and PGB04, the gene density with a single gene per 172 kbp and 94 kbp, respectively, is at least 10-fold lower than the overall gene density of the sequenced genomes of *Arabidopsis*, rice, poplar and grapevine (Table 1). The GC content (37%) of the two white spruce gDNAs was lower than the GC content of the rice genome (43.6%) and higher than those of the *Arabidopsis *(36%), poplar (33.7%), and grapevine (34.6%) genomes (Table 1) [30-33].

**Table 1:** General features of the gDNA sequences of the white spruce BAC clones PGB02 and PGB04 as compared to the genome sequence features of *Arabidopsis*, rice, poplar and grapevine.

	Genome Size (Mbp)	Predicted genes	Avg Gene length (bp)	Gene density (kbp per gene)	% TE	GC content (%)
*Arabidopsis thaliana*^1^	115	25,498	1,992	4.5	14.0	36.0
*Orzya sativa*^2^	389	37,544	2,699	9.9	34.8	43.6
*Populus trichocarpa*^3^	485	45,555	2,392	10.6	42.0	33.7
*Vitis vinifera*^4^	487	30,434	3,399	16.0	41.4	34.6
PGB02^5^	0.172	1	3,138	172	36.0	38.0
PGB04^5^	0.094	1	3,131	93.6	41.6	37.0

^1-4 ^[30-33]

^5^BAC insert size

### Analyses of the gDNA sequences for 3CAR and CYP720B4

**Figure 2:**
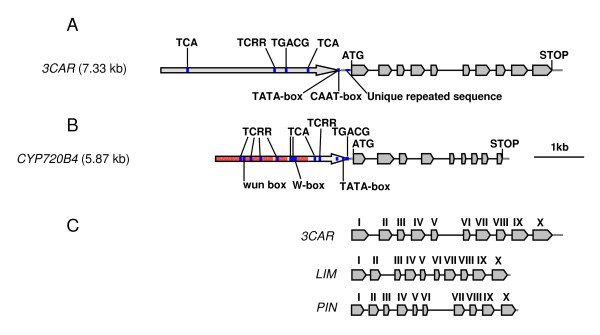
**Gene structure of white spruce 3CAR (A) and CYP720B4 (B) and comparison of 3CAR with the grand fir (*Abies grandis*) limonene synthase (LIM) and pinene synthase (PIN) genes (C)**. Exons of the 3CAR and CYP720B4 genes matching the cDNA sequences are shown with grey arrows separated by introns. The UTRs are shown with grey lines. ATG, start codon. Putative *cis*-acting elements were identified using the PlantCare database and positions are highlighted in blue (not to scale): wun-box, wound-responsive element (*Brassica oleracea*); W-box, fungal elicitor responsive element (*Petroselinum crispum*); TCRR, TC-rich repeats, *cis*-acting element involved in defence and stress responsivenes (*Nicotiana tabacum*); TCA, *cis*-acting element involved in salicylic acid responsiveness (*Brassica oleracea*); TGACG, *cis*-acting regulatory element involved in the MeJA-responsiveness (*Hordeum vulgare*). LIM, AF326518; PIN, AF326517; roman numbers in part C indicate conserved exons in 3CAR, LIM and PIN; the scale bar represents 1 kbp.

The genomic region of the 3CAR gene on PGB02 covers 3,541 bp, including a 198 bp 5'-UTR and 205 bp 3'-UTR which are part of the corresponding transcript isolated from cDNA (Figure 2A). The gene contains ten exons and nine introns, with intronic regions accounting for 35.4% of the gene sequence between the start and stop codon of this TPS gene. The genomic region of the CYP720B4 gene on PGB04 covers 3,131 bp over nine exons (1,452 bp) and eight introns and includes transcribed 5'- and 3'-UTRs of 38 bp and 134 bp, respectively (Figure 2B). The intronic region covers 50% of the gene sequence between the start and stop codon. The introns of 3CAR and CYP720B4 are of much lower GC content than the exons (% GC content exons/introns: 3CAR, 42.3/27.8; CYP720B4, 41.4/25.5).

### Analyses of upstream promoter regions of 3CAR and CYP720B4

Our analysis of upstream sequences for *cis*-regulatory elements covered 3,793 bp upstream of the ATG start codon for 3CAR and 2,500 bp upstream of the ATG start codon for CYP720B4. Putative *cis*-regulatory elements were identified by a similarity search of the PlantCARE database [34]. The region upstream of the ATG in 3CAR is unique until -3,973 bp which marks the location of a DNA transposon (Figure 1). In contrast, only the region from -1 bp to -749 bp upstream of the start codon of CYP720B4 is unique, followed by repetitive sequence (Figure 1 and Figure 2). Several promoter enhancing sequences (TATA and CAAT boxes) were identified in the region immediately upstream of the start codon of the 3CAR and CYP720B4 genes (Figure 2).

Since the spruce TPS and CYP720B genes are involved in the biosynthesis of defence related terpenoids induced by insects, pathogens, wounding or methyl jasmomate (MeJA) [21,35-38], we analysed the upstream genomic regions of 3CAR and CYP720B4 for putative *cis*-acting elements associated with plant defence responses (Figure 2). In CYP720B4, a conserved W box motif (TTGACC), which interacts in *Arabidopsis *with members of the WRKY transcription factor family to mediate responses to wounding or pathogen responses [39], is located at position -1,129 relative to the ATG of CYP720B4 on PGB04. A similar element (TGACG), involved in the MeJA-responsive gene expression in barley (*Hordeum vulgare*) [40], is detected at -1,266 relative to the start codon of 3CAR and at -79 relative to the start codon of CYP720B4. The upstream regions of 3CAR and CYP720B4 also contain a TCA-element at positions -815 and -3,291 in PGB02 and at positions -1,227, -676 and -1,162 (TCAGAAGAGG, GAGAAGAATA and CAGAAAAGGA) in PGB04, respectively. This element was first characterised as a *cis*-acting element involved in salicylic acid responsiveness and systemic acquired resistance in wild cabbage (*Brassica oleracea*) [41]. In addition, we identified several TC-rich repeats (ATTTTCTCCA) in the up-stream regions of 3CAR (one on PGB02) and CYP720B4 (six on PGB04). These sequences were previously described in tobacco (*Nicotiana tabacum*) as *cis*-acting elements involved in defence and stress responsiveness [42].

The upstream regions of the 3CAR and CYP720B4 genes also include a large number of putative transcription factor binding sites (37 for 3CAR; 19 for CYP720B4), implicated in light responsiveness in several other plant species. Interestingly, the promoter sequence including the transcribed 5'-UTR of the 3CAR gene on PGB02 contains a unique and conserved repeated sequence of 44 bp (TCAGGTTCTGCCATTGCCTTTTTAGTTCATTATCTTGAGCTGCC) which is located four times (with no more than two nucleotide changes) between -21 and -199 bp upstream of the start codon. Seventeen of the 44 bp in this repeated sequence have high levels (94-100%) of sequence identity to plant I-box transcription factor binding sites, which are involved in light responsiveness [43]. The actual role of this sequence in gene regulation is unknown, however, the prevalence of this sequence in the transcribed 5'-UTR of the 3CAR gene on PGB02 as well as in the 5'-UTR of two white spruce 3CAR-like ESTs (GQ03804.B7_I10 and GQ03313.B7_P23) and one Sitka spruce 3CAR-like EST (WS02910_I02) would make this sequence a relevant target for future transcription factor binding site analysis. In addition, several *cis*-acting elements previously identified in other plant species to be involved in responses to giberellin (GARE, TAACAGA; P-box; GCCTTTTGAGT), auxin (ARF, TGTCTC; TGA-element, AACGAC; AUX28, ATTTATATAAAT), ethylene (ERE, AWTTCAAA), and abiotic stresses (HSE, AAAAAATTTC; MBS, TAACTG; LTR, CCGAAA) were found in the upstream regions of 3CAR and CYP720B4.

### Identification and distribution of high and low complexity repeats in PGB02 and PGB04

Since repeat regions may offer a particular challenge for genome sequence assembly in conifers, it is important to accurately detect and mask high and low complexity repeats. A comparison of the PGB02 and PGB04 sequences with the genome sequences of *Arabidopsis*, rice, poplar, and grapevine [30-33] identified 3.7% of PGB02 and 3.0% of PGB04 with similarity (E-value < 10^-5^) to repetitive regions found in these angiosperms http://www.phytozome.net (Table 2). Using RepeatMasker [44] we found that high complexity repeats contribute to 21.9% and 17.6% of the sequence of PGB02 and PGB04, respectively (Table 2). We identified regions with similarity to RNA-based retroelements, predominantly Ty1/Copia and Gypsy/DIRS1 (long terminal repeat (LTR) element class) and a few segments of L1/CIN4 [long interspersed element (LINE) class] (Figure 1). In contrast to the large number of retroelement-based TEs, we found few regions (0.7% of total sequence of PGB02 and PGB04) with similarity to DNA-based transposons (*EnSpm*, *Helitron*, *MuDR *and *hAT*). Although PGB02 and PGB04 represent only a small fraction of the spruce gDNA, the identification of these DNA-based TEs is important as this is the first report of these elements in a gymnosperm.

**Table 2:** High complexity repeats in the white spruce gDNA of PGB02 and PGB04.

BAC	Repetitive sequences with similarities in angiosperms^1^	TEs detected with RepeatMasker^2^	Total repeat content^3^	Similarity to EST^4^ (%)
PGB02	3.7%	21.9%	36.0%	14.7%
PGB04	3.0%	17.6%	41.6%	17.1%

^1^Portion of the white spruce gDNA sequences of PGB02 and PGB04 with similarity to repeat regions identified in the genomes of *Arabidopsis*, rice, poplar and grapevine (cut-off E-value < 10^-5^); this excludes the coding regions of 3CAR and CYP720B4.

^2^Percentage of PGB02 and PGB04 sequences consisting of TEs as detected by the RepeatMasker using the *viridiplantae *section of the RepBase Update.

^3^Percentage of PGB02 and PGB04 sequences consisting of high complexity repeats as detected by pairwise comparisons of the two gDNA sequences.

^4^Fraction of the PGB02 and PGB04 sequences with similarity (at least 80 - 90% nucleotide sequence identity) to white spruce ESTs; this excludes the coding regions of 3CAR and CYP720B4; no EST hits were detected outside repeat regions.

While LTR retrotransposons have been reported in spruce with a high copy number, it is not known if members of the Ty1/Copia or Gypsy/DIRS1 families are active in spruce [27]. Presence of retrotransposons in the transcriptome and sequence conservation indicates that they are active. A BLAST search of the repetitive regions of PGB02 and PGB04 against EST databases (plant genome database, http://plantgdb.org/) yielded significant hits with ESTs from white spruce, Sitka spruce, interior spruce and Norway spruce as well as with pine species (Table 2). Pairwise comparison of the gDNA sequences of PGB02 and PGB04 revealed substantial sequence conservation within the repeat regions (Table 2). All regions with similarity to TEs reside in large, often continuous sections with high homology (average identity 86% over up to 3,000 bp) on PGB02 and PGB04 (Figure 1).

Screening for homologous regions between and within PGB02 and PGB04 also identified several previously undetected repeated elements, one of which represents a putative conifer specific repeat element (CSRE), which appears to have locally multiplied in PGB04 (Figure 1). A white spruce transcript with 91% identity to this CSRE is also present in the EST database (accession number WS0339.C21_N21). The occurrence of high complexity repeats in the BAC clones is estimated at 36.0% in PGB02 and 41.6% in PGB04, values which are substantially higher than those found in the fully sequenced genomes of *Arabidopsis *(10%) and poplar (12.6%), and similar to the genomes of rice (35%) and grapevine (38.8%) [30-33] (Table 2).

## Discussion

### Sequencing and assembly of BAC clones as a test for conifer genome sequencing

To date there is no sequence report for large segments of conifer gDNA, and researchers have avoided sequencing a conifer genome due to the large size and high content of repetitive elements. Several approaches are currently being considered for future efforts to sequence a conifer genome including the high-throughput sequencing of BAC libraries. To assess the feasibility of sequencing and assembling long, continuous segments of conifer gDNA, we targeted two white spruce defence genes, 3CAR and CYP720B4, for BAC clone isolation, sequencing and assembly. These genes were chosen because they are known to be members of large gene families with key functions in terpenoid biosynthesis.

Pre-assembled bidirectional reads of shotgun plasmid libraries for each BAC clone were assembled using PHRAP software resulting in a large number of contigs (15 for PGB02 and 14 for PGB04). Both BAC clones had areas of reduced quality reads with low or no sequence coverage bordered by regions of low complexity sequence repeats, which necessitated manual curation of the sequence assembly resulting in substantially improved sequence assemblies of two (PGB02) and one (PGB04) contigs. High complexity and simple repeats did not interfere with the automated PHRAP assembly and manual inspection of the contigs did not reveal falsely matched reads within the repeat regions. The use of pre-assembled paired reads and quality scores produced by PHRED balanced between tolerating discrepancies and complete mis-assembly of the data sets [45]. We found that most problems for automated sequence assembly resulted from chimeric clones in the plasmid libraries, bacterial DNA contamination, low-quality sequences and low-complexity repeats.

### Targeted BAC isolation of members of large conifer defence gene families provides insights into gene content of a conifer genome

The two genes targeted for BAC sequencing are members of large defence-related TPS and P450 gene families in spruce [20,46]. In the TPS gene family, members with more than 90% sequence identity can have distinct biochemical functions with non-overlapping product profiles [14,15]. In this study we demonstrate for the first time that it is possible to isolate, in an efficient and targeted fashion, BAC clones for specific members of the large conifer TPS and CYP720 defence gene families, thus providing new opportunities to characterize members of these important defence gene families at the genome level.

The 3CAR gene contains 10 exons and 9 introns, identical to the exon-intron structure of the grand fir (*Abies grandis*) monoterpene synthase genes (-)-limonene synthase and (-)-α/β-pinene synthase, previously cloned by PCR amplification of the gDNAs between the start and stop codons identified in the corresponding FLcDNAs (Figure 2C) [47]. The identity of the deduced amino acid sequence to the previously functionally characterised Norway spruce 3CAR [29] is 84%. The CYP720B4 gene has 9 exons and 8 introns, and is the first genomic structure reported for a gymnosperm P450 gene. A comparison of the CYP720B4 gDNA structure with the gDNA structures of *Arabidopsis *P450s shows highly conserved intron-exon boundaries between CYP720B4 and Arabidopsis CYP88, which is involved in the primary metabolism of giberellin biosynthesis. Both families of P450s share a similar reaction mechanism and catalyse consecutive oxidation steps of structurally similar substrates [21]. These findings suggest a common ancestor of CYP88 (primary metabolism) and CYP720B4 (secondary metabolisms).

Despite the large size of conifer genomes (estimated 20 to 40 Gbp; 200 - 400-fold larger than the genome of *Arabidopsis*), it is not likely that the spruce genome contains a proportionally larger number of protein coding genes than *Arabidopsis *as estimated from EST and FLcDNA discovery [3]. In contrast to previously sequenced angiosperm genomes, the spruce gDNA sequences of PGB02 and PGB04 reveal a low gene density, with a single gene per 172 kbp and 94 kbp respectively, which is at least 10-fold lower than the overall gene density of the genomes of *Arabidopsis*, rice, poplar and grapevine (Table 1). This observation of low gene density has also been confirmed by additional sequencing of several randomly selected spruce BAC clones (K. Ritland *et al.*, unpublished results).

In angiosperms, several mechanisms contribute to the expansion of gene families, including whole genome and chromosome segmental duplications [48], and tandem duplication of closely related genes [49]. For the gene family members targeted in this work, we did not find evidence for local tandem duplication.

### The upstream regions of 3CAR and CYP720B4 contain putative cis-acting elements consistent with the roles of these genes in induced defence

A large volume of previous research on the regulation and coordination of defence responses in spruce has targeted processes at the anatomical and molecular levels of induced metabolite accumulation, enzyme activities, and transcript abundance of genes involved in the biosynthetic pathways of terpenoid and phenolic defences [16,25,36,38,46,50-54]. In particular, 3CAR transcripts were up-regulated by real and simulated insect attack in Sitka spruce [36] and in Norway spruce [29]. In loblolly pine transcripts of the CYP720B4 related CYP720B1 were up-regulated in response to MeJA treatment [21]. In addition, large-scale proteome and gene expression profiling has identified putative transcription factors in spruce that were up-regulated in response to real or simulated insect attack [1,8,9]. This is the first report of the upstream sequences of conifer defence-related genes and the putative *cis*-acting elements located in those regions.

The upstream sequences of 3CAR and CYP720B4 each have more than five elements with sequence identity to *cis*-acting elements putatively involved in wound, stress and defence responses in angiosperms. The promoter region of the CYP720B4 gene is 95% to 99% identical with the corresponding PCR-amplified regions across several genotypes of Sitka spruce, hybrid interior spruce, and white spruce (data not shown). The conserved W-box motif present upstream of CYP720B4 is recognised and bound by transcription factors of the plant specific WRKY class which mediate pathogen defence responses in angiosperms [39]. More than 80 members of the WRKY family have been reported in pine [55,56] and more than ten different sequences with 60% to 80% identity to the *Arabidopsis *WRKY proteins AtWRKY6, AtWRKY3 and AtWRKY4, involved in defence, stress and pathogen responses [57,58] were found in the white spruce EST databases. These putative promoter regions and *cis*-acting elements represent valuable tools for future studies of the transcriptional regulation of conifer defence genes. Transformation of white spruce for characterization of promoters has been reported [59,60]. In future work we will use this transformation system, in parallel with transformation in heterologous plant systems, for functional testing of spruce TPS and P450 promoter constructs linked to reporter genes.

The finding of a novel 44 bp sequence element which is detected four times in the 5'UTR of the white spruce 3CAR gene on PGB02 was also found 19 times in the 5'UTR of the orthologous gene isolated as a cDNA in Sitka spruce. The conservation of this short sequence across spruce species suggests that this element has an important functional role in the regulation of the 3CAR gene.

### Genomic regions surrounding the 3CAR and CYP720B4 genes contain DNA and RNA based transposable elements

The genomic regions surrounding the 3CAR and CYP720B4 genes contain retrotransposons, DNA transposons and simple repeat sequences. With the exception of a fully preserved IS10 element present in the genomic sequence of PGB04 (likely the result of transposition from the bacterial host *E. coli *genome), all repetitive sequences appear to have accumulated a large number of mutations, deletions and rearrangements suggesting that these elements are no longer functional. The repeat regions in the gDNA of PGB02 (15%) and PGB04 (17%) have up to 89% similarity to white spruce TE-related ESTs. The presence of ESTs for these TEs indicates that members of these retrotransposon families may actively proliferate in conifers, potentially increasing genetic variability.

Remnants of DNA transposons of the cut-and-paste and copy-and-paste classes were found within 4 kbp and 500 bp of 3CAR and CYP720B4, respectively. In maize, the DNA-transposon *helitron *is associated with the duplication of CYP72A [61], and DNA-based transposons have been implicated in the capture and transduplication of host genes in rice, *Lotus japonicus *and *Arabidopsis *[62-64]. The proximity of DNA transposons to the protein coding 3CAR and CYP720B4 genes is consistent with the possibility that a DNA transposon-mediated translocation mechanism may contribute to the diversification of the conifer TPS and P450 gene families.

## Conclusion

We report the first sequence assembly and annotation of large segments of gDNA from a conifer. We also demonstrate that genomic BAC clones for specific members of large conifer defence gene families can be isolated in a very efficient and targeted fashion. This work provides important new information about the structure and content of conifer genome regions associated with the 3CAR and CYP720B4 genes in white spruce. Features of low gene density, high content of repetitive sequence regions, and richness of TEs identified in this work are likely characteristic of conifer genomes in general.

This work also provides relevant information for future efforts to sequence a conifer genome. Cost-efficiency is a critical factor in genome sequencing and is a function of sequencing chemistry, the complexity of the region being sequenced, and the quality of the assembly. Our simulation of the effect of BAC sequencing depth on assembly coverage showed that increasing the sequencing depth beyond 5 - 7 × coverage results in only a marginal improvement of the sequence assembly. The future sequencing of a conifer genome will likely use a combination of ultra-high throughput methods in combination with sequencing of BAC clones to anchor the high throughput reads. The bi-directional Sanger sequencing used in this study generated high quality sequences of more than 1,000 bp average length which were critical for the assembly of full-length BAC clones. Low quality reads resulting in poor sequence coverage occurred in regions of complex and simple repeats, which may also provide challenges for ultra high-throughput sequencing.

## Methods

### White spruce BAC library

Genomic (g)DNA was isolated from 200 g fresh weight of apical shoot tissue collected in April 2006 from a single white spruce (*Picea glauca*, genotype PG29) tree at the Kalamalka Research Station (British Columbia Ministry of Forests and Ranges, Vernon, British Columbia, Canada). A BAC library cloned into the *Hind*III site of pIndigoBAC-5 was made by BioS&T (http://www.biost.com/, Montreal). The non-arrayed library consisted of approximately 1.1 million BAC clones with an average insert size of 140 kbp, representing approximately 3× coverage of the white spruce genome.

### BAC library screening and shot-gun subcloning into plasmid libraries

The BAC library was screened by BioS&T for two target genes, a TPS gene encoding 3-carene synthase (3CAR) and a P450 gene encoding a diterpene oxidase (CYP720B4) using the procedures detailed in Isodore *et al. *[28]. In brief, the entire BAC library was plated (977 plates; approximately 1,200 colonies per plate) and colonies were transferred into ten 96-well plates with approximately 1,000 BAC clones per well (pool). Twenty super-pools of BAC clones were generated for each of the ten 96-well plates by combining the wells from twelve vertical rows and eight horizontal columns. These super-pools were screened by PCR for the two target genes. We used all available spruce EST and FLcDNA sequence information to design PCR primers that are, to the best of current knowledge, specific for the two target genes, while suppressing amplification of other known members of the spruce TPS and P450 gene families. Primers were designed to amplify fragments of approximately 500 bp, were evaluated with white spruce PG29 gDNA. The primer sequences (shown in 5'-3' orientation) are CTTTCAAGCCCAATACCCAAAGGCACTG and GGGAATGGCAATCACTGCATTGGTATAG for CYP720B4; and GGAGAATTAGTGAGTCATGTCGATG and CTCTGTCTGATTGGTGGAACAGGC for 3CAR. PCR products from super-pools were sequenced to confirm the identity of the target DNA. The individual pool (well containing the target gDNA clone) was identified, confirmed by PCR, and individual BAC clones isolated as described in Isidore *et al. *[28].

Isolated BAC clones PGB02 (3CAR) and PGB04 (CYP720B4) were digested with *Not*I to release the insert, and insert DNA size was determined by pulse field gel electrophoresis. The gDNA inserts of PGB02 and PGB04 were isolated by gel purification and sheared using a nebulizer (Invitrogen). After blunt-end repair, gDNA fragments were size fractionated on SeaPlaque agarose gels (CBM Intellectual Properties, Inc.). Fragments of 700 - 2000 bp were recovered and ligated into the *Sma*I site of pUC18. Plasmids were transformed in *E. coli *DH10B.

### Sequencing and automated sequence assembly

Shotgun subcloned plasmid libraries for PGB02 and PGB04 were arrayed in 384-well plates and gDNA inserts were Sanger-sequenced from both ends. Sequences were scanned and masked for vector sequences and contaminating bacterial sequences, eliminating 21.4% (PGB02) and 27.9% (PGB04) of the total sequences. This high level of contaminating DNA resulted from prolonged growth of bacterial cultures prior to BAC isolation. We have subsequently found that the use of Plasmid-Safe ATP-dependent DNase (Epicentre) reduces the amount of contaminating bacterial DNA.

Sequences were processed using PHRED software (version 0.020425.c) [65], quality-trimmed according to the high-quality contiguous region determined by PHRED, and vector-trimmed using CROSS_MATCH software http://phrap.org/. Vector and bacterial contaminated DNA sequences were identified by sequence alignments using megaBLAST to all UniVec and non-redundant bacterial sequences from NCBI respectively, and hits with 95% identity were subsequently masked with N's. Processed sequences were assembled with PHRAP http://www.phrap.org/ using the base quality files and with the bi-directional reads generated for each clone pre-assembled by PHRAP to match paired reads. The two commonly used assembling routines CAP3 and PHRAP were tested for their capability of assembling the BAC sequences. Despite CAP3 employing a higher stringency as compared to PHRAP [66], PHRAP assemblies of both BAC clones resulted in fewer but higher quality contigs which included more total sequences (PGB02: CAP3 49 contigs, PHRAP 14 contigs; PGB04: CAP3 19 contigs, PHRAP 14 contigs). The gDNA sequences identified in this work were submitted to NCBI GenBank under accession numbers FJ609174 (PGB02) and FJ609175 (PGB04).

### Manual curation of sequence assemblies

The contigs for PGB02 (15 contigs) and PGB04 (14 contigs) obtained by automated sequence assembly were manually curated. Sequences that prevented correct assembly such as sequences from chimeric DNA were removed and the remaining contigs were re-aligned. PGB02 was manually assembled into 2 contigs. Assembly of PGB04 into a single contig required the re-introduction of several sequences which had been previously identified as contaminating *E. coli *sequence. Examination of this *E. coli *sequence identified it as the insertion sequence (*EcIS10*) of the plasmid-associated bacterial transposon Tn10, which was presumably inserted into the BAC during proliferation. The left and right arms of the BAC vector (pIndigoBAC-5) were used to orient the remaining contigs, resulting in the final builds of PGB02 and PGB04.

Oligonucleotide primers were designed to bridge gaps in automated and manually curated sequence assemblies of PGB02. PCR using PGB02 BAC DNA and primers placed 1,112 bp and 993 bp on either side of the gap generated a single band of approximately 2.2 kbp. Sequencing of this PCR product verified up to 900 bp of sequence on either side of the gap but no additional sequence for the gap region were obtained, possibly due to low sequence complexity. For sequence finishing, oligonucleotide primers (shown in 5'-3' orientation) were designed based on the sequence scaffolds of PGB02 (AATTGGTCAATTCCTAAAACACCATG, AAATTATGGGTTTTAAGGGCTAGAGTTC) and PGB04 (AACAAATTTACTCATTTACCCGTGA, CCCATCAAAATCCATGCCCAAG, TTCCAAGTTCTTGTGGGAGGAG, GACTGATTTTCTCTCCACCAAGCAAG).

### Sequence analysis

Repetitive DNA was identified with the RepeatMasker software (A.F.A. Smit, R. Hubley & P. Green, unpublished data. Current Version: open-3.2.6 (RMLib: 20080801)), using the *viridiplantae *section of the RepBase Update [67] as a database. Gene models were predicted using the *ab initio *gene finder FGENESH (dicot matrix; [68]), Genscan and GeneMark.hmm with default parameters. Regions with similarity to DNA transposons were identified with RepeatMasker [44,67] with a threshold score over 200 and a length over 100 bp.

### Cloning and sequencing of up-stream regions of 3CAR and CYP720B4

The regions upstream of the start codon including the 5'UTR and promoter regions for 3CAR and CYP720B4 were amplified by PCR using white spruce PG29 gDNA as a template. Gene specific oligonucleotide primers (shown in 5'-3' orientation) were based on the BAC scaffolds of PGB02 (3CAR) (ACCCATCTTCACAAAATTAC, GTAGTCCATAACGAGCAGAA) and PGB04 (CYP720B4) (TGATATTTGGTCTGCCATGGGCG, CATTTCCCTGCATGTATTCAATGCC, CCACCACATAGTTAGACCGTGATGC).

## Authors' contributions

BjH, DH, MY, CIK and JB designed experiments, conducted the data analysis and interpretation of data and results. BjH, DH, CO and BrH carried out experiments. JB and KR conceived of the overall study. CR participated in the design of the study and coordination. BjH, DH, MY and JB wrote the manuscript. All authors read and approved the final manuscript.

## Supplementary Material

Additional file 1 **Figure S1 - Alignment of nucleic acid sequences of four closely related 3CAR gDNA fragments from white spruce (*Picea glauca*, Pg_3CAR1-4) and Sitka spruce (*Picea sitchensis*) (+)-3-carene synthase (Ps_Q09)**. The numbering above the alignment corresponds to the nucleotide position of the complete 3CAR gene of PGB02. Underlined sequences correspond to primer binding sites used for sequencing. Click here for file

Additional file 2 **Table S1**. Sequencing summary of plasmid libraries for PGB02 and PGB04. Click here for file

Additional file 3 **Figure S2 - Size and read allocation of the PHRAP assembled contigs of PGB02 (A) and PGB04 (B)**. The upper panel in each of A and B shows the number of reads in all contigs with the relative percentage of total reads given on top of the bars. The lower panel in A and B shows the length of all contigs given in bp with the relative percent of the length of the respective contig in percent of the total assembly given above the bars. Click here for file

Additional file 4 **Figure S3 - Effect of sequencing depth on assembly quality**. The sequence reads from five plates were used in all possible permutations to build assemblies corresponding to one, two, three and four combined plates. (A) The number of contigs and the number of nucleotides represented in the contigs. (B) Coverage relative to the manually curated sequence scaffold of PGB04 (93,592 bp). The fold coverage is indicated. Click here for file

Additional file 5 **Table S2**. Impact of sequencing depth on assembly quality of PGB04. Click here for file
