# Supplementary material for: Targeted isolation, sequence assembly and characterization of two white spruce (Picea glauca) BAC clones for terpenoid synthase and cytochrome P450 genes involved in conifer defence reveal insights into a conifer genome
Source: BMC Plant Biol. 2009 Aug 6;9:106. doi: 10.1186/1471-2229-9-106 (PMC2729077; doi:10.1186/1471-2229-9-106)
Supplement: Additional file 1 — Figure S1 - Alignment of nucleic acid sequences of four closely related 3CAR gDNA fragments from white spruce (Picea glauca, Pg_3CAR1-4) and Sitka spruce (Picea sitchensis) (+)-3-carene synthase (Ps_Q09). The numbering above the alignment corresponds to the nucleotide position of the complete 3CAR gene of PGB02. Underlined sequences correspond to primer binding sites used for sequencing. [file 1471-2229-9-106-S1.pdf]

```
          340                    360                    380                    400
Pg_3CAR1  ---------- ---------- ---------- ---------- ---------- CGATAACGTT GAACGATTGG GGATCGACAG 30
Pg_3CAR2  ---------- ---------- ---------- ---------- ---------- CGATAACGTT GAACGATTGG GGATCGACAG 30
Pg_3CAR3  ---------- ---------- ---------- ---------- ---------- CGATAACGTT GAACGATTGG GGATCGACAG 30
Pg_3CAR4  ---------- ---------- ---------- ---------- ---------- CGATAACGTT GAACGATTGG GGATCGACAG 30
Ps_Q09    GGAGAATTAG TGAGTCATGT CGATGATCTC CTTCAACACC TTTCGATGGT CGATAACGTT GAACGATTGG GGATCGACAG 80
          420                    440                    460                    480
Pg_3CAR1  ACATTTCCAA ACTGAGATCA AAGTTTCCCT CGATTATGTT TACAGGTCAG TGCTGACAGC CTTCAATTTT TTTTTATGTT 110
Pg_3CAR2  ACATTTCCAA GCTGAGATAA AAGTTTCCCT CGATTATGTT TACAGGTCAG TGCTGACAGC CTTCAATTTT TTTTTATGTT 110
Pg_3CAR3  ACATTTCCAA ACTGAGATAA AAGTTTCCCT CGATTATGTT TACAGGTCAG TGCTGACAGC CTTCAAATTT TTTTTATGTT 110
Pg_3CAR4  ACATTTCCAA ACTGAGATAA AAGTTTCTCT TGATTATGTT TACAGGTCAG TGCTGACGGC CTTCAAATTT TTGTTATGTT 110
Ps_Q09    ACATTTCCAA ACTGAGATCA AAGTTTCCCT CGATTATGTT TACAG----- ---------- ---------- ---------- 125
          500                    520                    540                    560
Pg_3CAR1  TTCACGACGA ATACACTTAA AACATAGAAA TTATTTTGGC CACCAGTT-A CTGGAGCGAA AAAGGCATTG GACCTGGGAG 189
Pg_3CAR2  TTCACGACGA ATACACTTAA AACATAGAAA TTATTTTGGC CACCAGTT-A CTGGAGCGAA AAAGGCATTG GACCTGGGAG 189
Pg_3CAR3  TTCACGACGA ATACACTTAA AACATAGAAA TTATTTTGGC CACCAGTTTA CTGGAGCGAA AAAGGCATTG GACCTGGGAG 190
Pg_3CAR4  TTCACGACGA ATACACTTAA AACATAGAAA TTATTTTGGC CACCAGTT-A CTGGAGCGAA AAAGGCATTG GATCTGGGAG 189
Ps_Q09    ---------- ---------- ---------- ---------- ------TT-A CTGGAGCGAA AAAGGCATTG GACCTGGGAG 158
          580                    600                    620                    640
Pg_3CAR1  AGATATTGTT TGTGCTGATC TCAACACCAC TGCCTTGGGG TTTCGAGTTC TTCGACTACA TGGATATACT GTGTTTCCAG 269
Pg_3CAR2  AGATATTGTT TGTGCTGATC TCAACACCAC TGCCTTGGGG TTTCGACTTC TTCGACTACA TGGATATACT GTGTTTCCAG 269
Pg_3CAR3  AGATATTGTT TGTGCTGATC TCAACACGAC TGCCTTGGGG TTTCGAGTTC TTCGACTACA TGGATATACT GTGTTTCCGG 270
Pg_3CAR4  AGATATTGTT TGTACTGATC TGAACACAAC TGCCTTGGGG TTTCGAATTC TTCGACTACA TGGATATACT GTGTTCCCGG 269
Ps_Q09    AGATATTGTT TGTGCTGATC TCAACACCAC TGCCTTGGGG TTTCGACTTC TTCGACTACA TGGATATACT GTGTTTCCAG 238
          660                    680                    700                    720
Pg_3CAR1  GTATCCGCAT TTTATTGATA AATCCCTCCT TTATTTTCTT TAATAAGGCA AC-------- ---------- ---------- 321
Pg_3CAR2  GTATCCGCAT TTTATTGATA AATCCCTCCT TTATTTTCTT TAATAAGGCA TCTGCGTGTA GATATTGATA GTTATATGTA 349
Pg_3CAR3  GTATCCACAT TTTATTGATA AATCACTCCT TTATTTTCTT TAATAAGGCA TC-------- ---------- ---------- 322
Pg_3CAR4  GTATCCACAT TTTATTGATA AATCACTCCT TTATTTTCTT TAATAAGGCA TC-------- ---------- ---------- 321
Ps_Q09    ---------- ---------- ---------- ---------- ---------- ---------- ---------- ---------- 238
          740                    760                    780                    800
Pg_3CAR1  ---------- ---------- TGCGTGTAGA TATTGATAGT TATATGTATT TTACGTTTAT GTCAGACGTG TTTGAACAAT 381
Pg_3CAR2  TTTTACGTTT ATGTCAGACG TGCGTGTAGA TATTGATAGT TATATGTATT TTACGTTTAT GTCAGACGTG TTTGAACAAT 429
Pg_3CAR3  ---------- ---------- TGCGTGTAGA TATTGATAGT TATATGTATT TTACGTTTAT GTCAGACGTG TTTGAACATT 382
Pg_3CAR4  ---------- ---------- TGTGTGTAGA TATTGATAGT TATATGTATT TTACGTTTAT GTCAGACGTG TTTGAACATT 381
Ps_Q09    ---------- ---------- ---------- ---------- ---------- ---------- -----ACGTG TTTGAACAAT 253
          820                    840                    860
Pg_3CAR1  TCAAAGACCA GATGGGGCGG ATTGCCTGTT CCACCAATCA GACAGAG 428
Pg_3CAR2  TCAAAGACCA GATGGGGCGG ATTGCCTGTT CCACCAATCA GACAGAG 476
Pg_3CAR3  TCAAAGACCA GATGGGGCGG ATTGCCTGTT CCACCAATCA GACAGAG 429
Pg_3CAR4  TCAAAGACCA GATGGGGCGG ATTGCCTGTT CCACCAATCA GACAGAG 428
Ps_Q09    TCAAAGACCA GATGGGGCGG ATTGCCTGTT CCACCAATCA GACAGAG 300
```
