# Supplementary material for: Targeted isolation, sequence assembly and characterization of two white spruce (Picea glauca) BAC clones for terpenoid synthase and cytochrome P450 genes involved in conifer defence reveal insights into a conifer genome
Source: BMC Plant Biol. 2009 Aug 6;9:106. doi: 10.1186/1471-2229-9-106 (PMC2729077; doi:10.1186/1471-2229-9-106)
Supplement: Additional file 2 — Table S1. Sequencing summary of plasmid libraries for PGB02 and PGB04. [file 1471-2229-9-106-S2.pdf]

Additional file 2: Table S1 - Sequencing summary of plasmid libraries for PGB02 and PGB04.

BAC	Number of plasmids sequenced	Contamination[1]	Number of sequence reads[2]	Number of contigs	Coverage[3]	Average plasmid insert length
PGB02	10x384	21.4%	6,954	15	15.6x	1,102bp
PGB04	5x384	27.9%	3,677	14	16.0x	1,138bp

[1]Contaminating sequences were identified using a BLAST search against the non-redundant nucleotide database at NCBI (blast.ncbi.nlm.nih.gov)

[2]The number of successful reads is given. Reads with masked repeats were assembled by PHRAP into contigs.

[3]The coverage is calculated from the number of non-redundant nucleotides and the physical size of both BAC clones.
