# Supplementary figures and images for: Targeted isolation, sequence assembly and characterization of two white spruce (Picea glauca) BAC clones for terpenoid synthase and cytochrome P450 genes involved in conifer defence reveal insights into a conifer genome
Source: BMC Plant Biol. 2009 Aug 6;9:106. doi: 10.1186/1471-2229-9-106 (PMC2729077; doi:10.1186/1471-2229-9-106)

# A

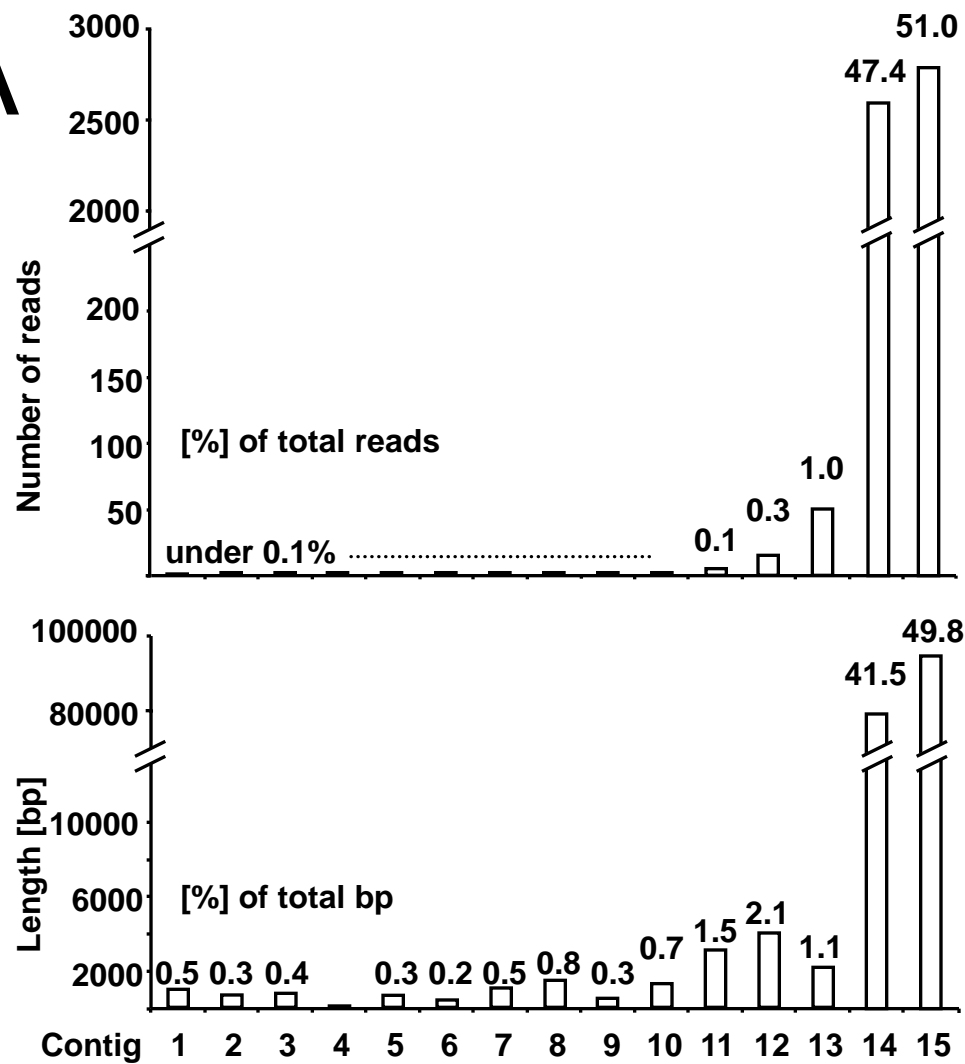

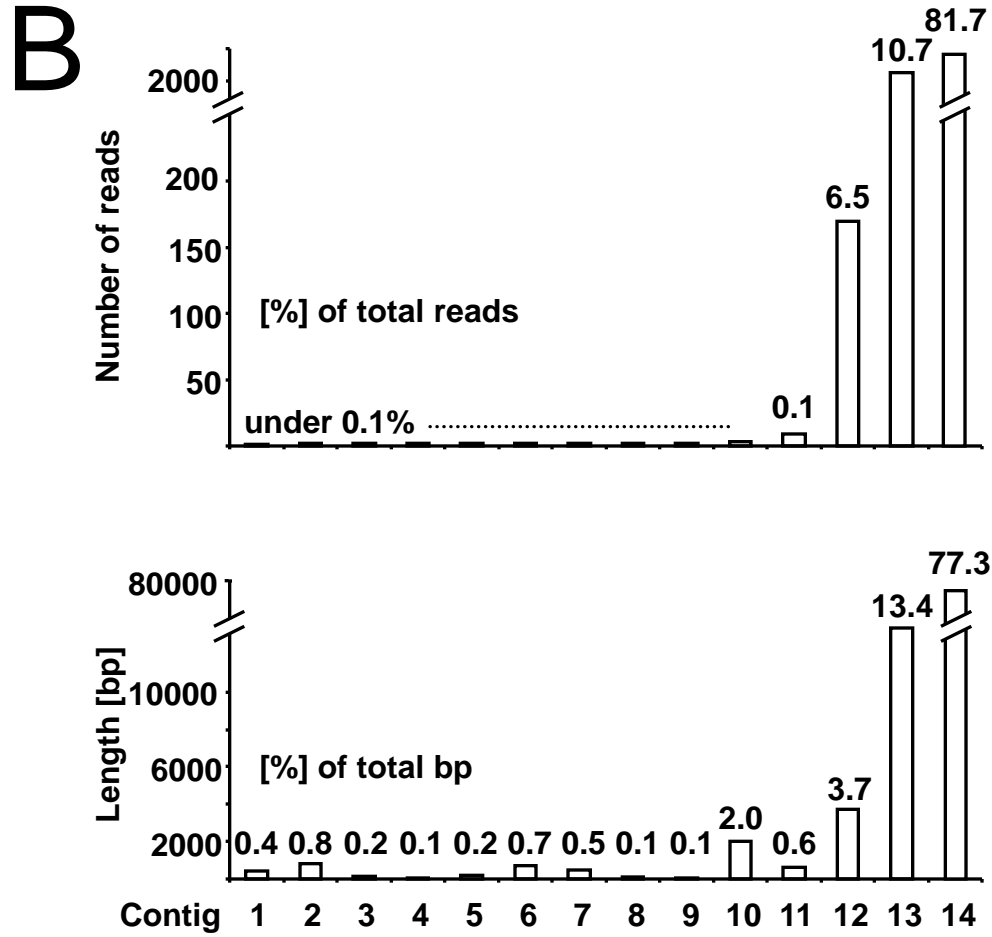

Figure S2

Supplement: Additional file 3 — Figure S2 - Size and read allocation of the PHRAP assembled contigs of PGB02 (A) and PGB04 (B). The upper panel in each of A and B shows the number of reads in all contigs with the relative percentage of total reads given on top of the bars. The lower panel in A and B shows the length of all contigs given in bp with the relative percent of the length of the respective contig in percent of the total assembly given above the bars. [file 1471-2229-9-106-S3.pdf]

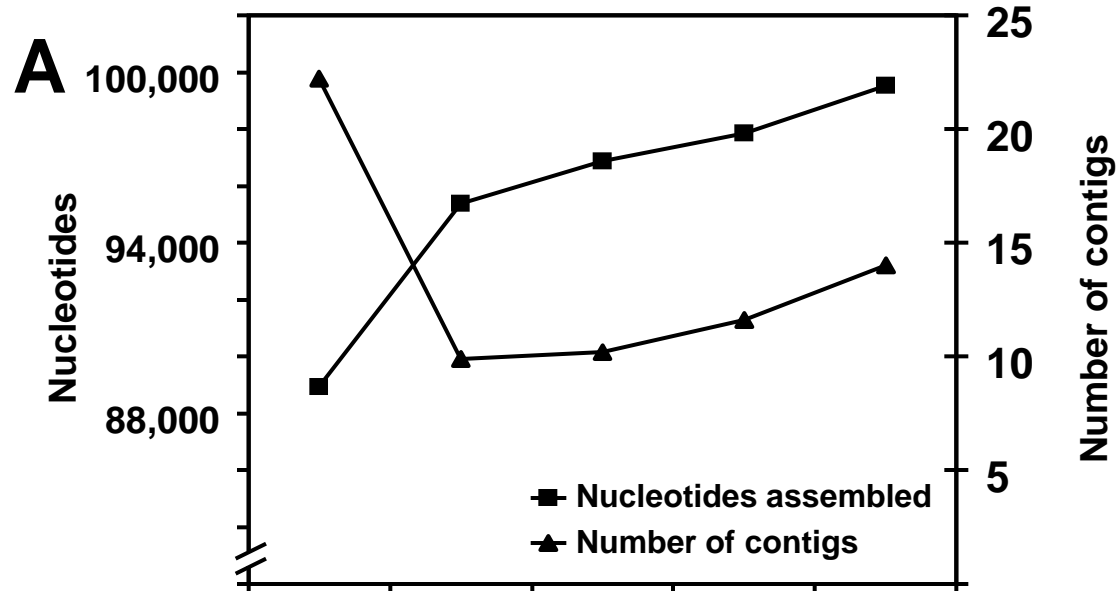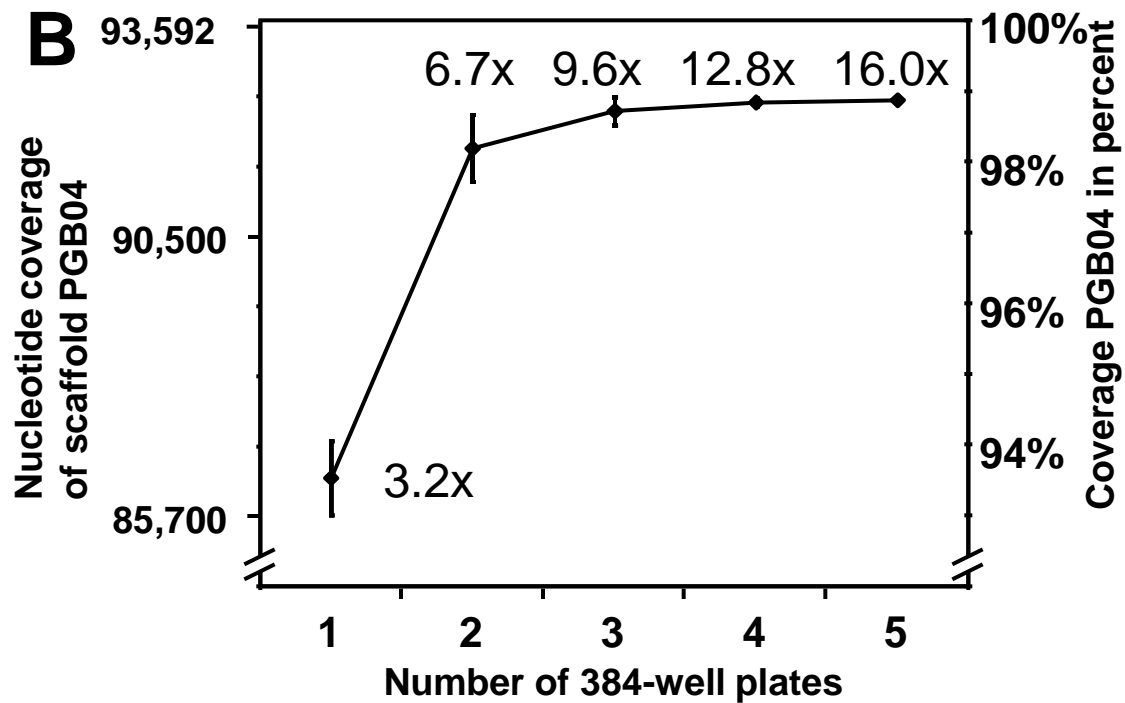

Supplement: Additional file 4 — Figure S3 - Effect of sequencing depth on assembly quality. The sequence reads from five plates were used in all possible permutations to build assemblies corresponding to one, two, three and four combined plates. (A) The number of contigs and the number of nucleotides represented in the contigs. (B) Coverage relative to the manually curated sequence scaffold of PGB04 (93,592 bp). The fold coverage is indicated. [file 1471-2229-9-106-S4.pdf]
