# Supplementary material for: Targeted isolation, sequence assembly and characterization of two white spruce (Picea glauca) BAC clones for terpenoid synthase and cytochrome P450 genes involved in conifer defence reveal insights into a conifer genome
Source: BMC Plant Biol. 2009 Aug 6;9:106. doi: 10.1186/1471-2229-9-106 (PMC2729077; doi:10.1186/1471-2229-9-106)
Supplement: Additional file 5 — Table S2. Impact of sequencing depth on assembly quality of PGB04. [file 1471-2229-9-106-S5.pdf]

Additional file 5: Table S2 - Impact of sequencing depth on assembly quality of PGB04.

Number of 384-well plates	1	2	3	4	5
Coverage of scaffold (bp)[1]	87,046	91,759	92,289	92,418	92,452
Coverage of scaffold (%)[2]	93.0	98.0	98.6	98.7	98.8
Average non-redundant (bp)[3]	299,266	598,532	897,797	1,197.063	1,496,329
Average total size (bp)[4]	88,929	95,376	96,860	97,847	99,515
Depth (fold)[5]	3.2	6.4	9.6	12.8	16.0

[1,2]The total number of nucleotides in contigs matching the final PGB04 scaffold (93,592 bp) is given in base pairs and percent.

[3]The sum of sequence lengths of all reads assembled into contigs is averaged. Nucleotides in overlapping regions of bidirectional reads were counted once.

[4]The sum of all contigs is given. The sum sometimes exceeds the size of the final PGB04 scaffold.

[5]Sequencing depth was calculated as a function of the average non-redundant base pair divided by the length of the scaffold PGB04.
